# Editorial for Special Issue “Fine Art Pattern Extraction and Recognition”

**DOI:** 10.3390/jimaging7100195

**Published:** 2021-09-29

**Authors:** Fabio Bellavia, Giovanna Castellano, Gennaro Vessio

**Affiliations:** 1 Department of Math and Computer Science, University of Palermo, 90133 Palermo, Italy; fabio.bellavia@unipa.it; 2 Department of Computer Science, University of Bari, 70125 Bari, Italy; giovanna.castellano@uniba.it

Cultural heritage, especially the fine arts, plays an invaluable role in the cultural, historical, and economic growth of our societies. Works of fine arts are primarily developed for aesthetic purposes and are mainly expressed through painting, sculpture, and architecture. In recent years, owing to technological improvements and drastic cost reductions, a large-scale digitization effort has been made, which has led to the increasing availability of large, digitized fine art collections. Coupled with recent advances in pattern recognition and computer vision, this availability has provided, especially researchers in these fields, with new opportunities to assist the art community by using automatic tools to further analyze and understand works of fine arts. Among other benefits, a deeper understanding of the fine arts has the potential to make works more accessible to a wider population, both in terms of fruition and creation, thus supporting the spread of culture.

The call for papers for the Special Issue “Fine Art Pattern Extraction and Recognition” was opened to anyone wishing to present advancements in the state of the art, innovative research, ongoing projects, and academic and industrial reports on the application of visual pattern extraction and recognition, for a better understanding and fruition of works of fine arts. The Special Issue solicited contributions from researchers in diverse areas such as pattern recognition, computer vision, artificial intelligence, and image processing. Furthermore, we also solicited the submission of papers as an extension of the works presented at the homonymous workshop we organized at the 25th International Conference on Pattern Recognition [1].

The Special Issue received several submissions, which underwent a rigorous peer review process. After the review process, 11 articles were selected based on the ratings and comments. The published articles cover various applications of cultural heritage and digital humanities research; focus on different branches fine arts such as painting, architecture, and photography; and develop and apply a range of techniques, from image processing to computer vision, based on handcrafted features and deep learning.

Artworks are subject to alterations over time due to various factors such as natural aging, external agents, inadequate conservation treatments, etc. Hence, techniques for monitoring, preserving, and restoring cultural heritage artifacts have become crucial. To this end, Daffara and Marini [2] present a non-destructive examination tool based on laser interferometry that uses laser speckle imaging for the effective mapping of subsurface defects in paintings. The system has been designed to be flexible, able to optimize its performance through an easy parameter adjustment. Trombini et al. [3] focused instead on the analysis and identification of color pigments using a digital camera, which served as a non-invasive, inexpensive, and portable tool for studying large surfaces. In their contribution, they propose a new supervised approach to camera characterization and color correction based on clustered data in which pigments are grouped based on their color or chemical properties. Fanfani et al. [4] focused on the restoration of historical photos, which offer valuable information and is an important source for art historians as they allow them to keep track of the changes that have occurred in a community and its living environment over time. To this end, they present a fully automatic method for the digital restoration of historical stereo photos, which exploits the redundancy of the content in stereo pairs to detect and correct defects in the original images while improving contrast and illumination. Amura et al. [5] focused on graphic documentation, which refers to the systematic collection of information derived from diagnostic investigation as well as the restoration and monitoring processes. To facilitate and improve this investigation, and to drastically reduce manual interventions, they propose a semi-automatic methodology aimed at generating an objective and accurate graphic documentation to plan restoration, monitoring, and conservation interventions.

In their contribution, Banfi and Mandelli [6] exploited computer vision in combination with drone photogrammetry to develop a digital model of the Arco della Pace monument in Milan for augmented reality applications. The proposed method can improve interactivity and the sharing of information between users and digital heritage models, as well as the accessibility of details that would not be visible from the ground (especially the sculpture that crowns the top of the building).

Other contributions have addressed the problem of high-level semantic analysis and the interpretation of artworks. With the advent of digitized art collections, such an analysis has acquired increasing importance as a means of providing new information within digital art image repositories, supporting both enthusiasts and experts in finding and comparing artworks. To this end, computer vision techniques are good candidates to aid in the automatic categorization and retrieval of artworks. Following this line of research, Pinciroli Vago et al. [7] experimentally compare several Class Activation Map techniques, which emphasize the areas of an image that contribute the most to the final classification performed by a convolutional neural network. This effort represents a step towards the creation of a computerized tool that is capable of highlighting variations in the positioning of iconographic elements, particularly for the detection of iconographic symbols in art images. Ghosh et al. [8] also focused on fine art classification, specifically proposing a method based on deep learning to classify geometric forms such as triangles and squares in mosaics. As a case study, a Roman mosaic is considered, which is digitally reconstructed by close-range photogrammetry based on standard photos. On the other hand, Tashu et al. [9] propose a multi-modal neural network based on sequential co-attention to classify artworks according to the emotions aroused in the observer. Emotion recognition in artworks is relevant as it can be used not only to group artworks, but also to provide recommendations that accentuate or balance a particular mood, or to find artworks of a specific style or genre that describe user-defined content in a user-defined affecting state. In her contribution, Cetinic [10] investigated the challenging problem of artwork captioning, which is the automatic generation of accurate and meaningful textual descriptions of artworks. To address this issue, she presents a captioning system developed by fine-tuning a transformer-based visual-language model. The results obtained suggest that it is possible to generate iconographically significant captions that capture not only the objects depicted, but also the historical and artistic context of an artwork. Abgaz et al. [11] also focused on the semantic enrichment of digitized cultural images and introduce a methodology for fully exploiting latent cultural information that is communicated visually by applying a combination of computer vision and semantic web technologies. A case study on food images is presented.

To support art historical research, Sindel et al. [12] did not focus on high-level concepts but on the distance measurement of the chain lines in historical prints, which constitute a sort of unique “fingerprint” of their paper structure. Since this process is typically manual, they propose an end-to-end trainable model based on a conditional generative adversarial network that performs line segmentation and parameterization in a multitask fashion.

We express our sincere gratitude to the authors for their contributions, to the reviewers for their efforts in reviewing the manuscripts, and to the editorial staff of the MDPI *Journal of Imaging* for their endless support in making this Special Issue possible. We hope it will benefit the scientific community and increase interest in this exciting area of research.
